# Cetuximab and radiation for primary and recurrent squamous cell carcinoma of the head and neck (SCCHN) in the elderly and multi-morbid patient: a single-centre experience

**DOI:** 10.1186/1758-3284-2-34

**Published:** 2010-11-26

**Authors:** Alexandra D Jensen, Zazie P Bergmann, Helena Garcia-Huttenlocher, Kolja Freier, Jürgen Debus, Marc W Münter

**Affiliations:** 1 Dept of Radiation Oncology INF 400 69120 Heidelberg, Germany; 2 Dept. of Head and Neck Surgery INF 400 69120 Heidelberg, Germany; 3 Dept. of Radiation Oncology Markus-Krankenhaus Wilhelm-Epstein-Str. 4, 60431 Frankfurt a. M., Germany; 4 Dept. of Oromaxillofacial Surgery INF 400 69120 Heidelberg, Germany

## Abstract

**Background:**

Chemoradiotherapy for head and neck cancer (SCCHN) is challenging in elderly, multi-morbid patients. Radioimmunotherapy (RIT) with cetuximab provides an option to enhance efficacy of radiotherapy without increased toxicity. We present a single-centre experience of RIT these patients.

**Methods:**

Toxicity and outcome was retrospectively analysed in patients treated with radiotherapy and cetuximab between 2006 and 2009. Treatment response was analysed at first follow-up, outcome was estimated using Kaplan-Meier analyses.

**Results:**

73 patients with primary/recurrent SCCHN were treated (re-irradiation: 22 patients). CTC grade 3 allergic reactions occurred in 4 patients, grade 3 acneiforme skin reactions leading to discontinuation of cetuximab in 3 patients.

Overall response rate was 59,4%, median locoregional and overall progression-free survival (PFS) was 18 and 15 months, overall survival (OS) 18 months.

**Conclusion:**

RIT is a feasible treatment option for elderly and multi-morbid patients with promising therapeutic activity. Long-term disease control can also be achieved in patients receiving RIT for re-irradiation.

## Background

Concurrent platin-based chemoradiotherapy has long been established as a standard in definitive treatment of squamous cell carcinoma of the head and neck (SCCHN) [1-3]. This applies to nasopharyngeal carcinoma [4,5], carcinoma of the larynx [6,7] or any other area of the head and neck [8,9]. Should the patient be unsuitable to undergo chemoradiotherapy, altered fractionation regimens provide a benefit over standard radiotherapy alone [10,11] in terms of local control and also overall survival [11]. However, there is a price to pay for higher local control rates: platin-containing regimens as well as altered-fractionation RT lead to higher rates of acute toxicity, i.e. mucositis, grade 3/4 leukopenia and therapy interruptions as compared to radiotherapy alone [4,6,10-12].

In 2006 though, Bonner and colleagues published results of combined radioimmunotherapy with the EGF receptor antibody cetuximab showing improved local control rates and overall survival without increase of toxicity or reduction in quality of life [13-15]. This trial has rapidly caused ample and animated discussions whether cetuximab should replace standard cisplatin in the treatment of SCCHN, given the fact, control rates were similar in retrospective comparisons with radiochemotherapy trials [16]. In the absence of direct or prospective randomised comparisons between the standard Cisplatin regimen and cetuximab in concomitant chemoradiation, guidelines still recommend using standard regimen for patients fit enough to undergo chemotherapy [17,18].

Although in principle, patients should receive curative therapy regardless of their age [19,20], elderly patients with SCCHN very often have multiple co-morbidities and/or poor initial performance status prohibiting intensified treatment schedules.

In accordance with the recommended use of RIT [17] and in-house standard procedures, these patients are offered RIT at our institution and have an option for combined therapy. This is a single centre experience with RIT using cetuximab for SCCHN from 2006 to mid-2009.

## Methods

Patients receiving radioimmunotherapy with cetuximab for stage III/IV or recurrent SCCHN between 01/2006 and 06/2009 were identified retrospectively from the hospital database. Baseline characteristics as well as treatment parameters were retrieved efficacy and toxicity of the combination regimen evaluated.

### Radiation therapy

#### RIT

According to our institutional protocols, target volumes were delineated in accordance with current guidelines and recommendations [21-23]. Primary RIT is aimed at delivering doses between 66 - 70 Gy to the primary tumour/involved nodes or tumour bed and between 54 - 57,6 Gy to the bilateral uninvolved neck.

If IMRT techniques were applied, integrated boost concepts were preferred applying 2.2 Gy/fraction to the primary/involved nodes and 1.8 Gy/fraction to the uninvolved neck. The median dose to the contralateral parotid gland was below 27 Gy, if possible, also the ipsilateral parotid gland was spared. The maximum dose to the spinal cord was limited to below < 40 Gy. 3D-RT usually employed sequential boost concepts at 2 Gy/fraction at similar target doses and organ constraints. In 2 D RT (conventional RT) the primary tumour/involved nodes or tumour bed were aimed at doses between 60 - 70 Gy, the uninvolved neck received 50 Gy at 2 Gy/fraction switching to nuchal, off-cord fields (6 MeV electrons) from 30 Gy. Commonly only patients in severely reduced performance state unable to tolerate longer treatment times were given conventional treatment; hence no concomitant boost concept was employed.

#### RIT as re-irradiation for local relapse

For patients who had already undergone a course of prior radiotherapy, the treatment volume was strictly limited to the gross tumour volume and did not include elective nodal levels. Doses were highly individualised but aimed at 50 - 60 Gy re-irradiation in 2 Gy/fraction [24] depending on elapsed time from the first course of RT and prior RT-dose.

#### Immunotherapy

After administration of anti-histamines (dimetindene) and corticosteroids (dexamethasone), cetuximab was administered as 400 mg/m2 body surface loading dose 7 days prior to RT-treatment start.

Weekly administrations of cetuximab 250 mg/m2 body surface followed for the duration of radiotherapy.

### Analysis

Treatment response was analysed 6 weeks post completion of RIT (first follow-up) according to RECIST criteria [25] based on available follow-up scans (CT or MRI) and clinical examinations. Treatment outcome (locoregional, distant and overall progression-free survival as well as overall survival) was evaluated using higher non-parametric statistics (Kaplan-Meyer survival analysis [26]/log-rank and Wilcoxon test) with the software Xlstat 2010. Progression-free survival was defined as the time from start of combined radioimmunotherapy until the first event (i.e. locoregional relapse, distant metastases, death). Similarly, overall survival was calculated from start of radioimmunotherapy until death from any cause. Toxicity was assessed using CTC v 3.0.

## Results

Seventy-three patients with SCCHN (median age 69 a (42 - 86)) were treated with radioimmunotherapy (RIT) using cetuximab, all of these patients received RIT instead of chemoradiation due to poor overall performance status and multiple co-morbidities (Table 1).

**Table 1:** Reasons for RIT

Reasons for RIT	Pts
Liver cirrhosis	9
COPD	4
Cardiac co-morbidities	18
Renal impairment	12
Prior TPF induction	4
Poor performance score (ECOG > 2)	35
Within palliative systemic therapy	2

Fifty-one patients received RIT as part of their primary treatment (group 0) or treatment for local relapse but did not have prior radiation therapy (group 1). Twenty-two patients underwent re-irradiation for disease relapse (group 2), one of these patients continuing cetuximab for systemic disease management while undergoing RT for bone metastases, hence this case was omitted for further analysis. Median follow-up (f/u) was 10.1 months (0.17 - 43.6), 12 patients were lost to f/u, and 32 patients had deceased as of 05.06.2010 (Table 2).

**Table 2:** Baseline characteristics

SCCHN Localisation		
laryngeal carcinoma	12 pts	
hypopharyngeal carcinoma	6 pts	
oropharyngeal carcinoma	21 pts	
carcinoma of the oral cavity	19 pts	
other localisations	15 pts	
		
**Grading**		
G1	2 pts	
G2	28 pts	
G3	31 pts	
not available	12 pts	
		
**RT**		available for analysis
primary treatment	51/73 pts	48 pts
re-irradiation	22/73 pts	21 pts
		
**age**		
median	69 a	[42 - 86]
mean	68 a	
		
**follow-up**	(months)	
median	10,1	[0,17 - 43,6]
mean	13,8	
lost to f/u	12/73 pts	
		
**deceased**		
total	32 pts	
primary RT	18 pts	
re-RT	14 pts	

Patients received a median dose of 66 Gy [16 - 70,6 Gy] for definitive RT (Table 3) and a median dose of 45 Gy [34,2 - 59,6 Gy] for re-irradiation corresponding to a median cumulative lifetime dose of 106,4 Gy [96 - 125,6 Gy] (Table 4). The median interval between first and second course of irradiation was 83,5 months [17 - 293 mo], these patients either had an R2 resection after salvage surgery or were medically unable to have any major surgical intervention. Most patients were treated by IMRT (primary RIT: 28/51 pts, re-RT: 16/22 pts). Patients undergoing 3 D or conventional treatment could not tolerate longer treatment durations per day hence were treated with less complex techniques. All of the patients had marked co-morbidities prohibiting combined radiochemotherapy and therefore received combined radioimmunotherapy with cetuximab with a median of 6 cycles [1-9].

**Table 3:** Adverse reactions under RIT (all patients)

°III allergic reaction leading to discontinuation of cetuximab	4 pts (5,6%)
Skin reactions leading to cetuximab breaks	1 pt (1,4%)
Skin reactions leading to discontinuation of cetuximab	3 pts (4,2%)
Overall °III skin reactions	6 pts (8,3%)
Discontinuation of treatment due to detoriation of overall condition	5 pts (6,9%)

**Table 4:** Radioimmunotherapy for primary/recurrent SCCHN (no prior RT)

tumor stages		
**T1**	8 pts	
**T2**	8 pts	
**T3**	3 pts	
**T4**	30 pts	
		
**N0**	15 pts	
**N1**	4 pts	
**N2**	31 pts	
**N3**	0 pts	
		
**primary RT**		
median dose (Gy)	66	[16 - 70,6]
IMRT	28 pts	
3D	9 pts	
conventional RT	9 pts	
		
**RIT**		
		
first treatment	38/51 pts	
		
RIT for disease relapse	13/51 pts	
> 1 cycle cetuximab	48/51 pts	
		
		
**response (48 pts) at first follow-up**		
**CR**		10 pts
**PR**		19 pts
**SD**		2 pts
**PD**		5 pts
**dna**		5 pts
**f/u unavailable**		7 pts

Four patients showed grade 3 allergic reactions to cetuximab at first exposure leading to discontinuation of the drug, therefore these patients were not included in further response/survival analysis. Grade 3 acneiforme skin reactions were observed in 6 patients (8,3%) and leading to discontinuation of cetuximab in 3 patients (4,2%). Five patients (6,9%) had to discontinue RIT due to deterioration of overall condition (Table 5). Grade 4 or 5 skin reactions were not observed.

**Table 5:** Radioimmunotherapy for recurrent SCCHN receiving re-RT

Re-RT (21 pts, 1 pt treated for bone metastases)				
IMRT	16/22 pts			
3D-RT	3/22 pts			
conventional RT	3/22 pts			
**prior RT**		**Dose (Gy)**	**time between RT treatments (months)**	**cumulative Dose (Gy)**
	**median**	66	83,5	106,4
	**min**	40	17	96
	**max**	70,4	293	125,6
			
**Re-RT**				
	**median**	45		
	**min**	34,2		
	**max**	59,6		
			
**response (21 pts)**				
**CR**		2 pts		
**PR**		10 pts		
**SD**		2 pts		
**PD**		5 pts		
**dna**		1 pt		
**f/u unavailable**		1 pt		

Re-RT resulted in 6 cases of grade 3 late toxicity: 4 cases of permanent feeding tube dependency (2 cases due to oesophageal stenosis/dysphagia, 2 cases due to silent aspiration). One patient developed painful fibroses within the re-RT field and one patient developed mandibular joint fibrosis necessitating surgical intervention. No patient within the RIT cohort however, has shown any signs of tissue necrosis, osteoradionecrosis, or radiogenic vascular haemorrhage (3 patients died of tumour bleeding due to progressive disease).

According to RECIST, we found an overall response rate (PR and CR according to RECIST) of 59.4% (41/69 pts), which was similar both in primary RIT (60,4% (29/48 pts)) and RIT for re-RT 57% (12/21 pts) (Table 3 and 4).

**Figure 1:**
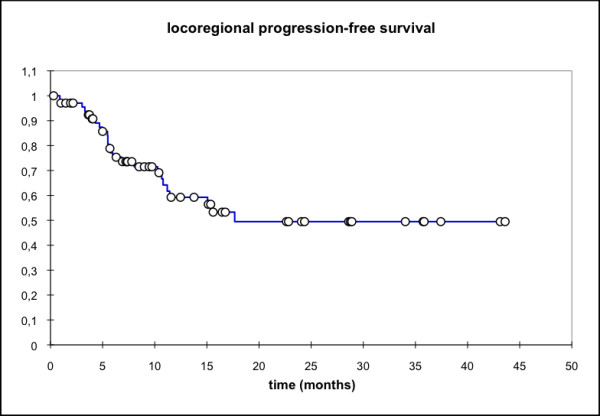
**Locoregional PFS, all pts (n = 69); Mean 25.6 months, 95% CI: 20.4 - 30.8 months**.

**Figure 2:**
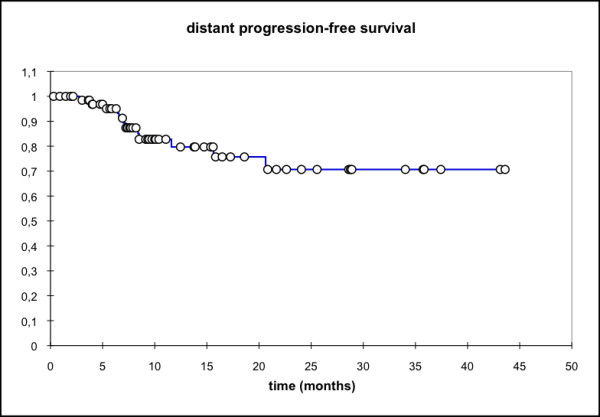
**Distant PFS, all pts (n = 69); Mean 29.6 months; 95% CI: 25.5 - 33.7 months**.

**Figure 3:**
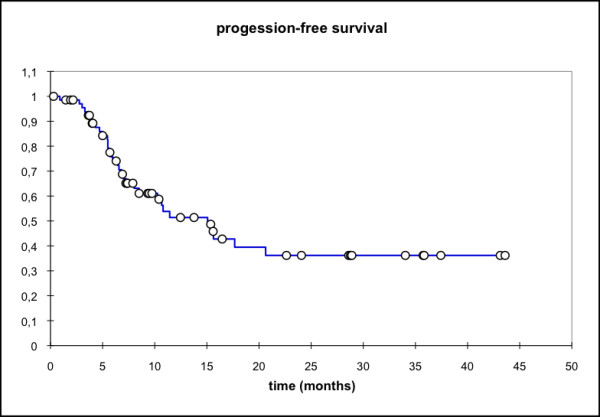
**Overall PFS, n = 69 pts; Mean 21.3 months; 95% CI: 16.3 - 26.3 months**.

**Figure 4:**
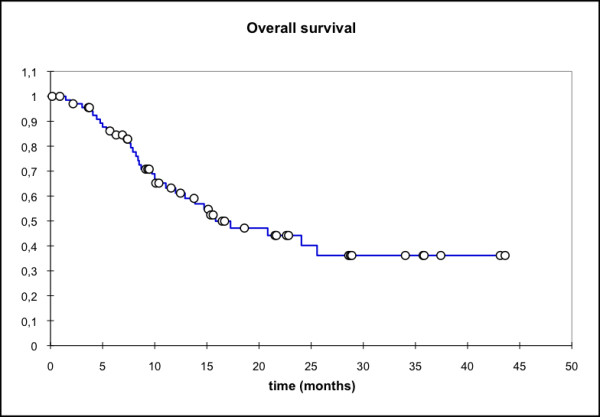
**Overall survival, n = 69 pts; Mean 23.1 months; 95% CI: 18.4 - 27.7 months**.

Median locoregional progression-free survival (PFS) (69 pts) was 17.7 months (mean 25.6 mo, 95% CI: 20.4 - 30.8 mo) while the median distant PFS is not reached yet (mean 29.6 mo, 95% CI: 25.5 - 33.7 mo). The median PFS (overall) is 15.1 months (mean 21.3 mo, 95% CI: 16.3 - 26.3 mo) and the median overall survival in this patient cohort was 15.8 months (mean 23.1 mo, 95% CI: 18.4 - 27.7 mo) (Figure 1, 2, 3, 4).

**Figure 5:**
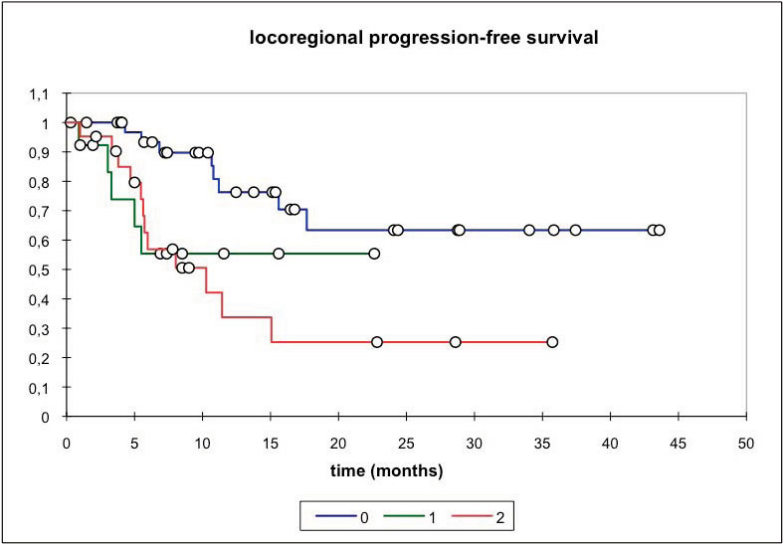
**Locoregional PFS**. 0: RIT as primary therapy; mean 31.5 months, 95% CI 24.4 - 38.6 months. 1: RIT for disease relapse; mean 8.0 months; 95% CI 5.4 - 10.7 months. 2: RIT as re-RT; mean 12.8 months; 95% CI 7.6 - 17.9 months. p = 0.009 (0 vs. 1); p = 0.001 (0 vs 2); p = ns (1 vs 2)

**Figure 6:**
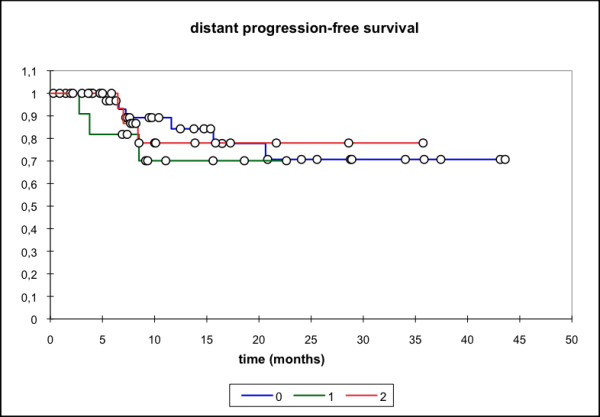
**Distant PFS**. 0: RIT as primary therapy; mean 30.2 months, 95% CI 24.7-35.7 months; 1: RIT for disease relapse; mean 12.5 months; 95% CI 8.9 - 16.2 months. 2: RIT as re-RT; mean 23.9 months; 95% CI 18.2 - 29.7 months. no statistically significant difference between the groups

**Figure 7:**
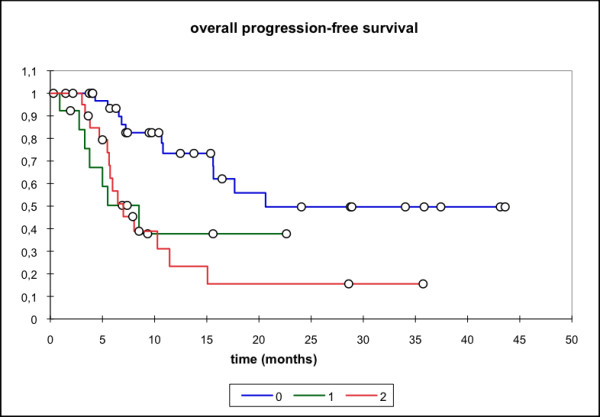
**Overall PFS**. 0: RIT as primary therapy; mean 24.8 months, 95% CI 19.0-30.6 months 3. 1: RIT for disease relapse; mean 8.7 months; 95% CI 5.2 - 12.3 months. 2: RIT as re-RT; mean 8.6 months; 95% CI 6.5 - 10.6 months. p = 0.0014 (0 vs 1); p = 0.001 (0 vs 2), p = ns (1 vs 2)

**Figure 8:**
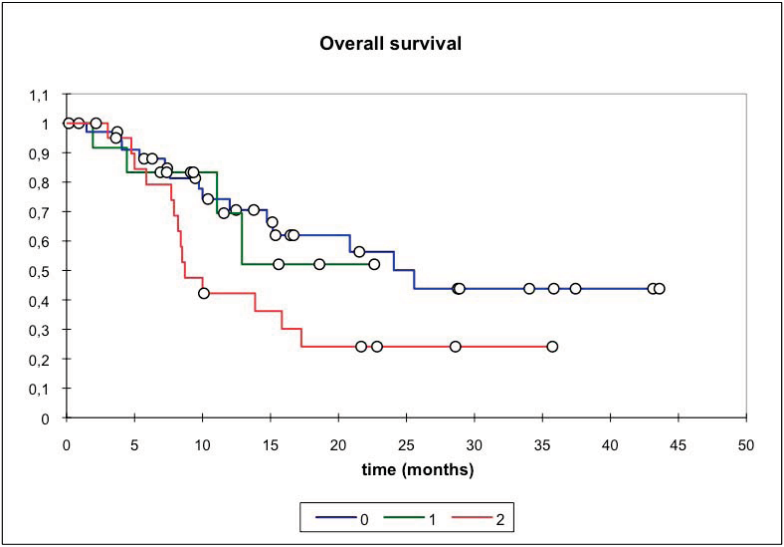
**Overall survival**. 0: RIT as primary therapy; mean 26.5 months, 95% CI 20.1 - 33.0 months. 1: RIT for disease relapse; mean 14.0 months; 95% CI 9.9 - 18.1 months. 2: RIT as re-RT; mean 12.5 mo; 95% CI 9.2 - 15.7 months. p = 0.039 (0 vs 2), p = ns (0 vs 1 and 1vs 2)

Subgroup analysis (group 0: primary treatment, group 1: treatment for relapse but no prior RT, group 2: re-RT) showed significantly lower locoregional and overall PFS for the two latter groups with median locoregional PFS not reached for group 0 (mean 31.53 mo, 95% CI: 24.43 - 38.63 mo), group 1 (mean 8.0 mo, 95% CI: 5.4 - 10.7 mo)/group 2 (median locoregional PFS 10.3 mo, 95% CI: 5.6 - 15.1 mo). Similar results were seen for the median overall PFS (group 0: 20.1 mo, group 1: 8.5 mo, group 2: 7 mo,). Median OS was also significantly higher in group 0 (primary treatment) when compared to group 2 (p = 0.039) (median 25.6 mo vs 8.7 mo in the re-RT group) (Figure 5, 6, 7, 8).

Whenever possible, patients received IMRT treatment for their SCCHN, however, in our analysis we did not observe a significant impact of treatment technique on locoregional PFS, overall PFS or OS (data not shown).

## Discussion

Guidelines recommend application of potentially curative treatment regimen to patients independent of age [19,20]. However, special considerations must be made for elderly patients with co-morbidities associated with a higher incidence of treatment-related complications.

Our patient cohort consisted of elderly and co-morbid patients with a median age of 69 years where chemoradiation was deemed prohibitive and curative surgery was either not possible due to the patients' condition or refused by the patient.

In view of the patients' poor overall condition at treatment start, aggressive new treatment regimens such as induction chemotherapy followed by radiation [27,28] were also no option in this patient cohort. In order to still offer curative treatment to these patients, radioimmunotherapy was administered based on the assumption of lower rate of treatment-related side effects as compared to radiochemotherapy [13,14].

Although the rate of allergic reactions at first exposure is slightly higher than reported by Bonner and colleagues, we found combination treatment with cetuximab was generally well tolerated leading to a discontinuation of the medication in only 3 patients. Major acute side-effects (apart from the 3 allergic reactions) were not observed, notably there were no CTC °4/5 acute effects. Even though, 5 patients did discontinue treatment for various reasons (3 patients terminating treatment by their expressed wish, 2 patients due to infections leading to deterioration of overall condition). However, this did not seem to be correlated to cetuximab in particular, rather to underlying initial/pre-therapeutic poor condition and advanced disease. Overall, the combination treatment seems feasible in this subset of patients.

The overall remission rate seen in our patients was 59.4% translating into a median PFS of 18 months (overall) [21 months for patients receiving RIT for primary treatment, 8.3 and 6.7 months for patients receiving RIT for locoregional relapse/re-irradiation] and a locoregional PFS of 15 mo (overall) [not reached for primary treatment/treatment for local relapse; 10 mo for re-irradiation] respectively. These are somewhat lower than remission rates and local control found by Bonner or larger chemoradiotherapy trials [9,29]. However, about half of our patients had very advanced tumours (T4: 30 pts) and nodal stages (N2: 31 pts).

Second, we also included patients with locoregional relapse or multiple relapses of their SCCHN following initial surgical management as well as patients receiving re-irradiation for unresectable or only partially resectable disease.

Third, all our patients had a very limited to poor pre-therapeutic performance status and advanced age. As demonstrated by Cooper et al [30] and more recently by Agarwal and Siddiqui et al. [31,32] one of the most important predictive factors for patient outcome are tumour and nodal stage as well as initial performance status [33] and also advanced age. Hence, our patients did in fact show a very negative pre-selection with a combination of adverse prognostic factors not normally represented in prospective and/or randomised clinical trials.

The median overall survival (overall 16 months, group 0 24 months, group 1, group 2 8.7 months) is still higher than obtained by RT-only in randomised-controlled radiochemotherapy trials [8,9,29,34]. Despite the higher median age hence a priori lower life expectancy, the OS is even higher in our patients receiving RIT for primary treatment (group 0). Median locoregional PFS with 18 months (overall) is also promising with the median locoregional PFS above 23 months (group 1) and 43 months (group 0) and not yet reached to date.

Almost one third of our patients also received RIT for re-irradiation. While our re-irradiation doses as well as cumulative lifetime doses were comparatively conservative [35], treatment response and long-term disease control is possible also in this subset of patients. Our response rates compare favourably with response rates reported by Lee et al [36] and De Crevoisier et al. [37]. Long-term control is possible even though macroscopically complete surgical salvage in our patients was either not possible or not attempted at all. Together with advancing age and multiple recurrences prior to re-RT been proven negative prognostic factors [36,37]. Re-irradiation with high cumulative doses usually comes at the cost of increased late toxicity. Unfortunately, the follow-up of this patient subgroup with a median overall survival of 8.7 moths is probably too short to detect further potential late effects. It should also be mentioned that only 6 cases of grade 3 therapy-associated late toxicity were noted (feeding tube dependency: 4 pts, painful fibroses: 1 pt, mandibular joint fibrosis: 1 pt). Hence the rate of ≥ grade 3 late toxicity is comparable to late toxicity rates reported by Janot et al [24]/Sulman et al [38] and even lower than reported by Salama et al [35] or Roh et al. [39]. It is probably noteworthy that IMRT was used for primary and re-irradiation for maximum normal tissue sparing whenever tolerable for the patient.

Most of the RT treatments were carried out using IMRT with only few patients treated with other techniques. A significant influence of RT technique on outcome could not be shown unlike the findings reported by of Clavel et al [40].

## Conclusion

Results from the RTOG recursive partitioning analysis [30] as well as Agarwall et al [31] and Yulut-Caloglu et al [38] suggest initial pre-treatment performance score as well as advanced age as an independent prognostic factor in the treatment of head and neck patients. Our retrospective analysis demonstrates that RIT is a feasible and well tolerated treatment option in these patients and that Cetuximab can be safely administered as part of radioimmunotherapy to patients whose overall performance status or co-morbidities do not allow combined radiochemotherapy. Combination treatment with Cetuximab shows promising therapeutic activity with respect to response rates, local control and overall survival in the elderly, multi-morbid and extensively pre-treated patient population. Also, we can support the assumption that patients treated for local relapse of their disease or undergoing re-irradiation for recurrent SCCHN show worse outcomes as compared to the primary situation; however, local control can still be achieved in this subset of patients.

## Competing interests

Prof. Jürgen Debus is a member of the Merck KGaA advisory board. All other authors declare that they have no competing interests.

## Authors' contributions

All authors read and approved the final manuscript
